# Structural insight into FANCI–FANCD2 monoubiquitination

**DOI:** 10.1042/EBC20200001

**Published:** 2020-07-29

**Authors:** Landing Li, Winnie Tan, Andrew J. Deans

**Affiliations:** 1 Genome Stability Unit, St. Vincent’s Institute of Medical Research, Fitzroy, Victoria 3065, Australia; 2 Department of Medicine (St. Vincent’s Health), The University of Melbourne, Fitzroy, Victoria 3065 Australia

**Keywords:** biochemistry, DNA synthesis and repair, enzyme activity, Fanconi anemia, protein structure, ubiquitin ligases

## Abstract

The Fanconi anemia (FA) pathway coordinates a faithful repair mechanism for DNA damage that blocks DNA replication, such as interstrand cross-links. A key step in the FA pathway is the conjugation of ubiquitin on to FANCD2 and FANCI, which is facilitated by a large E3 ubiquitin ligase complex called the FA core complex. Mutations in FANCD2, FANCI or FA core complex components cause the FA bone marrow failure syndrome. Despite the importance of these proteins to DNA repair and human disease, our molecular understanding of the FA pathway has been limited due to a deficit in structural studies. With the recent development in cryo-electron microscopy (EM), significant advances have been made in structural characterization of these proteins in the last 6 months. These structures, combined with new biochemical studies, now provide a more detailed understanding of how FANCD2 and FANCI are monoubiquitinated and how DNA repair may occur. In this review, we summarize these recent advances in the structural and molecular understanding of these key components in the FA pathway, compare the activation steps of FANCD2 and FANCI monoubiquitination and suggest molecular steps that are likely to be involved in regulating its activity.

## Introduction

Fanconi anemia (FA) is a genetic disorder that has intrigued scientists and illuminated medical discoveries for at least half a century. The first patient to ever receive a curative bone marrow transplant from umbilical-cord blood had FA [1], the first documented case of pre-implantation embryo selection to create a matched donor was in FA [2], and the first successful trials of gene therapy for a bone marrow failure syndrome were recently successfully completed in patients with FA [3]. The first discovered breast cancer predisposition genes, BRCA1 and BRCA2 also turned out to be FANC genes, named FANCS and FANCD1 [4,5]. The ‘FA pathway’ – the assembly of 22 FANC gene products working together in a common process of DNA repair – turns out to be critical for gene editing by CRISPR [6], the movement of transposases throughout the genome [7,8], crossing over during meiosis [9], and the resistance of cells to chemotherapy drugs [10,11]. The study of FA has directed many fields of research.

But one area where FA has trailed discoveries in other fields is in structural biology. As such, FA investigators have lacked the ability to fully interrogate hypotheses, examine the direct effect of specific mutations on the function of a given FANC protein, or even know the key role of many of the 22 FANC gene products. Many bizarre ideas about certain FANC proteins have appeared in a flourish and then promptly disappeared; ideas that could have easily been put to rest by an investigation of the protein structure concerned. One critical constant in our understanding of FA has been the knowledge that central to the ‘FA pathway’ is monoubiquitination of FANCD2, and its partner protein FANCI.

Known since 2001, FANCD2 monoubiquitination (the covalent ligation of a single molecule of the 8.5 kDa protein ubiquitin) occurs during ongoing DNA replication and is increased after DNA damage [12]. In particular, FANCD2 (and FANCI) monoubiquitination leads to retention of the complex at sites of stalled replication. FA cells (that all lack FANCD2 monoubiquitination) are exquisitely sensitive to agents that increase stalled replication, such as interstrand cross-links or R-loops [13,14]. At least nine proteins (FANCA, FANCB, FANCC, FANCE, FANCF, FANCG, FANCL, FAAP20 and FAAP100) make up a so-called ‘FA core complex’ [15]. Mutations in the genes that encode FA core complex proteins or FANCD2 and FANCI, represent the defect in over 97% of FA [16]. The FA core complex is a ubiquitin E3 ligase that monoubiquitinates the FANCI–FANCD2 substrate complex at the site of DNA damage [17]. It is thought that this recruits downstream nucleases containing ubiquitin binding domains to repair the interstrand DNA cross-links [18,19]. DNA binding of FANCI–FANCD2 is required for monoubiquitination and activation of the FA pathway [17,20], however it was unclear how this activates DNA repair.

Here, we will discuss how recently described structures of the FA core complex, FANCI–FANCD2 and monoubiquitinated FANCI–FANCD2 have finally addressed the ‘structural deficit’ in our understanding of FA [21–25]. We will suggest mechanistic steps for FANCI–FANCD2 monoubiquitination and raise new questions and opportunities for further discovery.

### Structure of FANCI–FANCD2 heterodimer

The FA pathway converges on the FANCI–FANCD2 heterodimer. Not only it is a substrate for the FA core complex, but also a potential platform for the recruitment of downstream DNA repair proteins including FAN1 nuclease, and other FANC proteins (FANCD1, J, N, O, and P) [26,27]. The crystal structure of mouse FANCI–FANCD2, uncovered in 2011 (PDB: 3S4W, [28]) revealed that FANCI and FANCD2 have solenoidal structures that are surprisingly similar to each other, despite the proteins sharing only 14% conservation.

**Figure 1:**
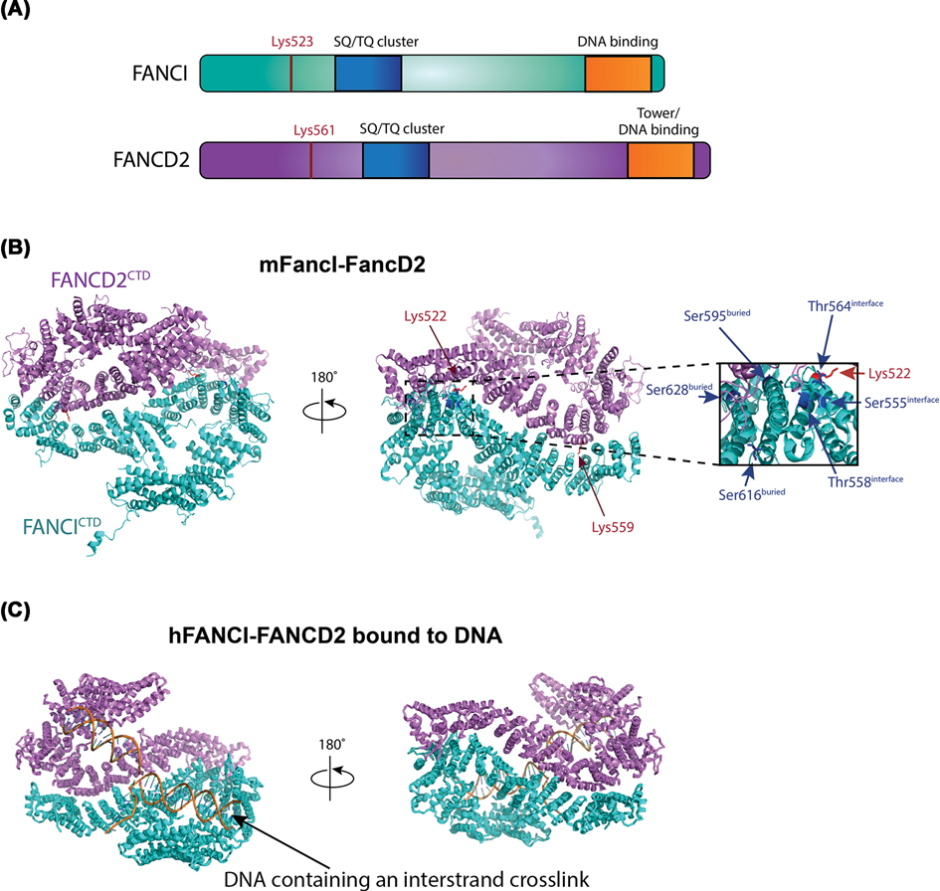
Overall structural comparisons of mFancI–FancD2 and hFANCI–FANCD2 bound to interstrand cross-linked DNA (**A**) The same domain architecture is shared by FANCI and FANCD2 and is shown schematically above. (**B**) Structure of mouse (m)FancI–FancD2 (PDB ID: 3S4W) where the ubiquitination sites, Lys^559^ residue of FANCD2 and Lys^522^ residue of FANCI are shown in red sticks; FANCI phosphorylation sites are shown in blue sticks. Close-up view of FANCI phosphorylation sites, Ser^555^, Thr^558^ and Thr^564^ exposed on the heterodimer interface, and Ser^595^, Ser^616^ and Ser^628^ residues buried in the complex. (**C**) Structure of human (h)FANCI–FANCD2 bound to interstrand cross-linked DNA (PDB ID: 6VAA). FANCD2 is colored in violet, FANCI in cyan and DNA in orange. Abbreviation: CTD, C-terminal domain.

The heterodimer forms a pseudo-symmetric structure, reminiscent of two antiparallel saxophone-like shapes. Side-on it adopts a trough-like shape, within which single and double-strand DNA binding sites were predicted based upon a lower resolution structure of FANCI:DNA complex in the absence of FANCD2 [28]. Unexpectedly, the monoubiquitination sites of FANCD2 (K559 in mouse corresponding to K561 in human) and FANCI (K522 in mouse corresponding to K523 in human) – were buried in the FANCI^NTD (N-terminal domain)^-FANCD2^NTD^ interface (Figure 1A,B) [28]. The crystal structure revealed little about how the heterodimer is a substrate for the FA core complex, or how it might activate the next steps in repair but did reveal the location of several key phosphorylation sites in FANCI. In particular, the ATR-kinase substrate sites in FANCI, S555, T558 and T564 are exposed on a surface adjacent to the FANCD2 interface. In cells, the integrity of these sites is essential for FANCD2 monoubiquitination [29], and *in vitro* their phosphorylation or phosphomimic mutation leads to stabilization of the complex on DNA [30].

The molecular details of FANCI–FANCD2:DNA interactions were resolved in the cryo-electron microscopy (EM) structure of FANCI–FANCD2 bound to interstrand cross-linked double-stranded DNA (dsDNA) (PDB: 6VAA, average resolution 3.8 Å) (Figure 1C) [25]. The structure of FANCI–FANCD2 revealed that dsDNA runs though the heterodimer [25], consistent with biochemical studies that indicate dsDNA is required for monoubiquitination to occur [17,31]. Interestingly, the monoubiquitination sites remain buried in the complex, which retains the same open trough-like shape as apo FANCI–FANCD2. The previously observed preferential binding of FANCI to DNA [28,32] suggests that the tight binding of FANCI to DNA acts as an ‘entry point’ for FANCI–FANCD2 binding to stalled replication forks. Both FANCI and FANCD2 C-terminal domains (CTDs) are required for DNA binding within the ID2 complex [21,25]. This finding is consistent with previous low-resolution cryo-EM structure of FANCI–FANCD2 showing a C-terminal ‘Tower’ DNA-binding domain in FANCD2 [20].

**Figure 2:**
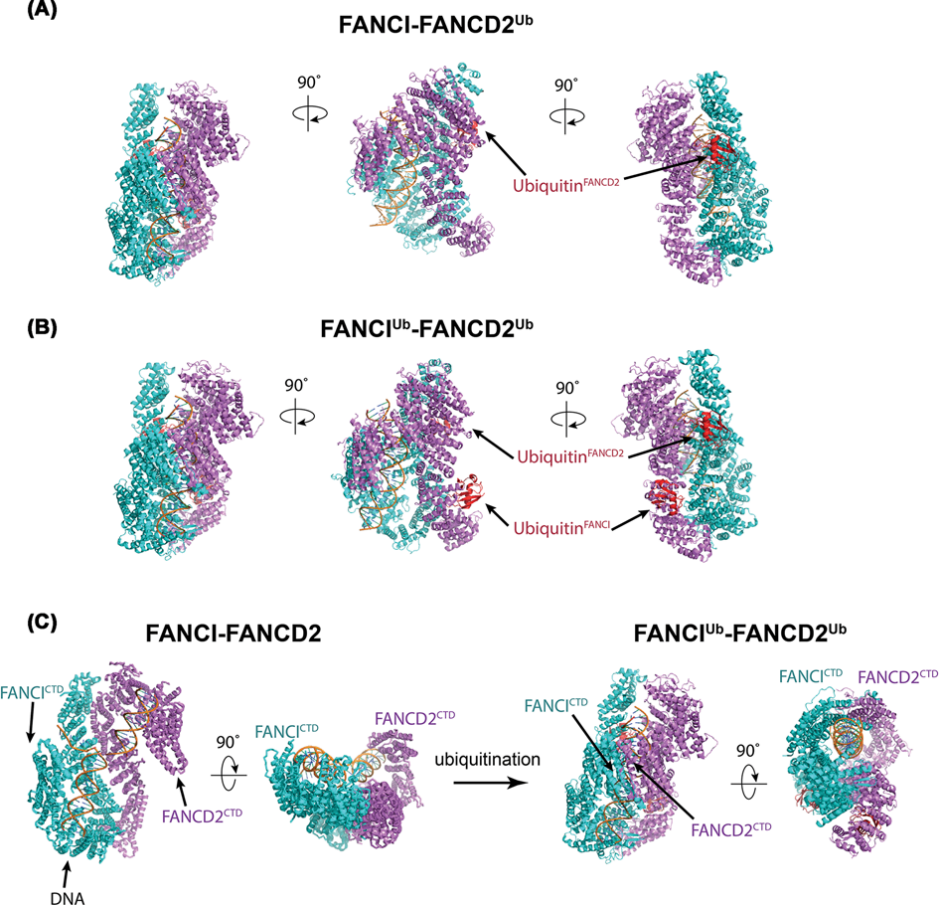
Overall structural comparison of FANCI–FANCD2, FANCI–FANCD2^Ub^ and FANCI^Ub^–FANCD2^Ub^ (**A**) Structure of singly monoubiquitinated human FANCI–FANCD2^Ub^ (PDB ID: 6VAF). (**B**) Structure of dually monoubiquitinated human FANCI ^Ub^–FANCD2 ^Ub^ (PDB ID: 6VAE). (**C**) Comparison of FANCI–FANCD2 (PDB ID: 6VAA) and di-ubiquitinated FANCI–FANCD2 (PDB ID: 6VAE). FANCD2 is colored in violet, FANCI in cyan, ubiquitin in red and DNA in orange.

**Figure 3:**
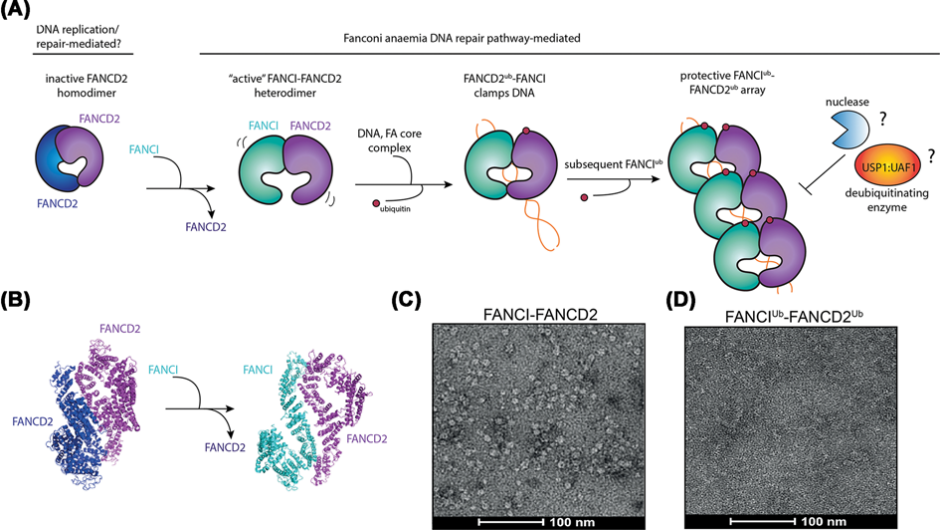
Proposed activation mechanism of FANCI^Ub^–FANCD2^Ub^ filamentous array (**A**) FANCD2 forms an inactive homodimer which is inaccessible to DNA binding. FANCI displaces one of the FANCD2 and assembles into the FANCI–FANCD2 heterodimer, which is the substrate for FA core complex E3 ligase and DNA binding. FANCD2 ubiquitination clamps FANCI–FANCD2 heterodimer on DNA, which further stimulates ubiquitination of FANCI to form protective array against DNA degradation by nucleases or deubiquitination by USP1:UAF1. (**B**) Comparison of FANCD2 homodimer (PDB ID: 6TNI) and apo-FANCI–FANCD2 heterodimer (PDB: 6VAD). (**C**) Negative-stained electron micrograph of apo-FANCI–FANCD2. (**D**) Negative-stained electron micrograph of di-ubiquitinated FANCI–FANCD2 filamentous arrays. FANCD2 is colored in blue or violet, FANCI in cyan, ubiquitin in red and DNA in orange.

So why is FANCI–FANCD2 complex a heterodimer and not a homodimer of just FANCD2? Another recent structure study revealed that FANCD2 homodimers can exist, when the protein is purified in the absence of FANCI [21]. A cryo-EM structure of isolated FANCD2 (PDB: 6TN1, average resolution 3.4 Å) revealed an overall closed trough-like structure that is not associated with DNA [21,25] (Figures 2A,B and 3B). We hypothesize that because the ‘entry point’ of FANCI:DNA interaction is absent, the ubiquitination sites of FANCD2 remain buried in the FANCD2^NTD^–FANCD2^NTD^ interface. Further, DNA cannot enter between the two subunits in any other way, because the normal channel between FANCI^CTD^ and FANCD2^CTD^ is blocked by FANCD2^CTD^–FANCD2^CTD^ association. It is possible that FANCI first binds to DNA and FANCD2 prior to ubiquitination. This finding explains why FANCD2 alone is such a poor substrate for monoubiquitination by the FA core complex [17] and suggests there might be dynamic equilibrium of FANCI–FANCD2 heterodimer and FANCD2 homodimer in cells that could act as a regulatory mechanism in the pathway.

### Structure of monoubiquitinated FANCI–FANCD2 reveals DNA clamping

Several ground-breaking studies of FANCI–FANCD2 in the ubiquitinated state have significantly advanced our understanding of the role that ubiquitin plays when conjugated on to the complex. The cryo-EM structures of *Gallus gallus* (chicken) and human monoubiquitinated FANCD2 bound to non-ubiquitinated FANCI have been resolved at 4.1 Å (gFANCI–gFANCD2^Ub^, PDB: 6TNF [21]) and 3.5 Å (hFANCI–hFANCD2^Ub^, PDB: 6VAF [25]) resolution, respectively. These structures uncovered a ubiquitination-induced rotation of FANCD2 by 70°, which brings FANCD2 closer to FANCI to grip the dsDNA [21] (Figure 2A). Metaphorically, this rotation closes the trough observed in apo-FANCI–FANCD2, clamping the complex on DNA. This conformational change significantly decreases the off-rate of FANCI–FANCD2 to dsDNA [21,24], which is the central event for DNA repair activation. The main finding of the present study is that the ubiquitin acts as a wedge to stabilize the FANCI–FANCD2 closed conformation on DNA [21].

Four concurrent studies also demonstrated a requirement for FANCD2^Ub^ but not FANCI^Ub^ in DNA clamping, for human, chicken and *Xenopus* homologs [21,24,25,33]. The cryo-EM structures of di-monoubiquitinated human FANCI–FANCD2 (FANCI^Ub^–FANCD2^Ub^, PDB: 6VAE, 3.8 Å) reveals why this is the case (Figure 2B) [25]. Importantly, FANCI^Ub^ barely alters its conformation compared with FANCI (Figure 2C). Comparison of FANCI–FANCD2 and FANCI^Ub^–FANCD2^Ub^ reveals that by far the majority of movement is in FANCD2. FANCD2 monoubiquitination changes the conformation of FANCD2, and there is a rotation of both FANCI and FANCD2 subunits, which brings their CTD toward each other [25].

An entirely new protein:protein interaction interface is created by these huge conformational changes [21,25]. Strikingly, the original interface between the two proteins now becomes surface exposed, and an entirely novel interaction interface is established. First, a new N-terminal association of FANCI and FANCD2 is established by the rotation of FANCD2 by 59′, and a sliding of 15′ relative to FANCI. The original NTD interface of the apo structure now presents a surface for ubiquitin binding on the opposite molecule. Although ubiquitin is now exposed on the surface of FANCI or FANCD2, the hydrophobic patch of ubiquitin consisting of Ile^44^ and Val^70^ residues required for binding of ubiquitin binding domain is buried within the FANCI–FANCD2 complex [21,25]. This precluded FANCI–FANCD2 binding by other proteins with ubiquitin-binding domains (such as UBZ and CUE domains) [34–36]. Furthermore, biochemical pulldown of monoubiquitinated FANCI–FANCD2 with DNA repair proteins that contain ubiquitin-binding domains showed that monoubiquitination did not alter interaction of FANCI–FANCD2 with the DNA repair proteins [24].

This new interface created by ubiquitination rationalizes a proposed mechanism by which deubiquitination is controlled. FANCD2^Ub^–FANCI is preferentially deubiquitinated compared with FANCD2^Ub^–FANCI^Ub^ [17,33], even though the two structures are essentially identical. The only main difference is the occlusion of the USP1 interaction site in FANCD2 [37] by the presence of the ubiquitin now located on the opposing FANCI [25,33]. A patient associated mutation in FANCI (R1285Q) may cause this protective mechanism to break down, as FANCI R1285Q mutant displayed a two-fold faster deubiquitination of FANCD2 [25]. In cells, unloading of FANCD2^Ub^–FANCI^Ub^ may be instigated by DVC1-p97 [38] but removal of DNA from the FANCD2^Ub^–FANCI^Ub^ complex *in vitro* is sufficient to again permit the activity of USP1:UAF1 on the complex [17,33] highlighting the critical role that DNA plays in stabilizing the structure of the heterodimer. Importantly, the structural changes with-respect to DNA suggest that FANCD2^Ub^–FANCI^Ub^ complex will lose its structure-specific binding activity compared with FANCI–FANCD2, which initially has preference for branched DNA molecules [25]. This finding suggests several new avenues of investigation.

### What is the function of clamping FANCI–FANCD2?

The burying of the hydrophobic binding patches of ubiquitin against the FANCI–FANCD2 heterodimer creates an interesting conundrum: it means that some other conformational change to the complex would be necessary for ubiquitin-binding DNA repair proteins to be recruited. This is a possibility but has not yet been observed in any of the studies described above. Further, we recently demonstrated that purified FANCI^Ub^–FANCD2^Ub^ is not bound preferentially by any of the DNA repair proteins that had been hypothesized to be important in the FA pathway [24]. This includes nucleases such as FAN1 and SLX4 [18,19]. So, if ubiquitination is not required for recruiting DNA repair cofactors, then what is the exact role of FANCI–FANCD2 ubiquitination? We propose that DNA clamping protects the DNA or controls its branch migration, by forming arrays on dechromatinized DNA at stalled replication forks. Evidence for this comes from our striking observation of long filamentous arrays of FANCI^Ub^–FANCD2^Ub^ when it is ubiquitinated and purified together with long dsDNA (plasmid length compared with 20–30 bp in the cryo-EM structures) and observed under the electron microscope (Figure 3C,D) [24]. This was unexpected. Such arrays were not observed in DNA bound, but unmodified FANCI–FANCD2, nor in previous EM studies of human or frog FANCI–FANCD2 [20,39]. But the formation of large domains of FANCI^Ub^–FANCD2^Ub^ may explain extensive accumulation of FANCD2 at site-specific DNA damage sites of kilobases (kb) to megabases (mb) in length [40]. FANCI^Ub^–FANCD2^Ub^ arrays may protect newly synthesized replication forks from attack by nucleases, and explain why FA cells see extensive degradation of stalled replication forks of equivalent kb–mb intervals [41]. Singly ubiquitinated FANCI–FANCD2^Ub^ may be a precursor for di-ubiquitinated FANCI–FANCD2 filament but this hypothesis has not been tested.

Wang et al. offer a modified model [25]. They propose that FANCI^Ub^–FANCD2^Ub^ complex may act as a sliding DNA clamp on DNA duplex, similar to PCNA. PCNA forms a ring around DNA that slides together with the replicative polymerases, to increase their processivity during replication [42]. The observation that FANCI^Ub^–FANCD2^Ub^ (and also FANCI–FANCD2^Ub^) fully encloses DNA, and loses its substrate specificity led to the suggestion that it might also slide. Using different DNA molecules, Wang et al. demonstrated that dissociation of the complex from linear DNA is ∼16-fold faster than FANCI^Ub^–FANCD2^Ub^ to circular DNA [25]. As such, it is possible that FANCI^Ub^–FANCD2^Ub^ slides off the end of linear DNA, but could possibly be blocked by a barrier such as an R-loop or an interstrand cross-link – two structures that are known to induce accumulation of FANCD2 monoubiquitination on DNA [13,14]. As such, the blockage of one sliding FANCI^Ub^–FANCD2^Ub^ complex could promote a filamentous structure, as all the other FANCI^Ub^–FANCD2^Ub^ molecules accumulate behind it.

Based on all these ideas, we propose that FANCI–FANCD2 function is regulated through the following steps: (1) FANCI forms a heterodimer with FANCD2 upon DNA damage; (2) FANCD2 is monoubiquitinated by the FA core complex and the heterodimer is clamped on DNA; (3) in the absence of immediate USP1:UAF1-mediated deubiquitination, FANCI is monoubiquitinated by the FA core complex leading to FANCD2 being protected from deubiquitination; (4) ubiquitination clamps further FANCI–FANCD2 molecules on DNA to form protective filamentous array (Figure 3A). An architectural model of FANCI–FANCD2 arrays in the future will help determine whether they form a thermodynamically favored filamentous structure, or are merely forced into proximity by the clamping action of monoubiquitination.

### Structure of the FA core complex

In FA, the majority of causative mutations occur in subunits of the FA core complex [16], which catalyze the monoubiquitination of FANCD2 and FANCI. Two recent breakthrough studies have determined the high-resolution EM structure of the FA core complex [23,43]. At the molecular level, the FA core complex consists of nine FANC subunits: FANCA, FANCB, FANCC, FANCE, FANCF, FANCG, FANCL, FAAP100 and FAAP20 [17].

**Figure 4:**
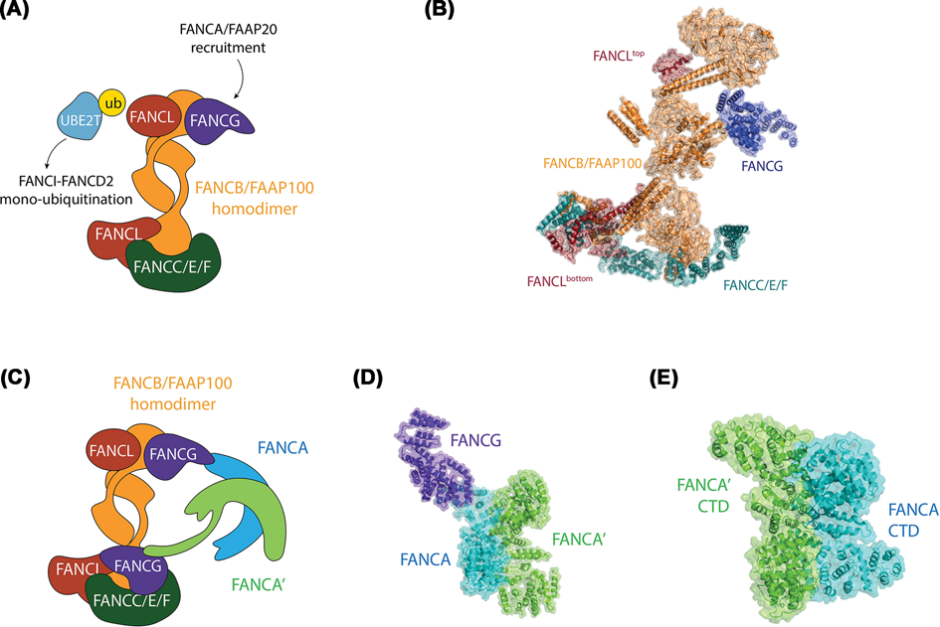
Structure of asymmetric FA core complex and FANCA–FANCG subcomplex (**A**) Schematic of the chicken FA core complex. The arrows indicate potential protein:protein interactions of FANCA/FAAP20, UBE2T E2 enzyme and FANCI–FANCD2. (**B**) Structure of chicken FA core complex (PDB ID: 6SRI). (**C**) Schematic of the human FA core complex, colored by protein(s). FANCB/FAAP100 – orange; FANCL – red; FANCG – purple; FANCC/E/F – cyan; FANCA – cyan; FANCA′ – lime; FANCG and FANCG′ – purple. (**D**) Structure of FANCA–FANCG complex (PDB ID: 6LHV). (**E**) Structure of FANCA CTD dimer (PDB ID: 6LHS).

The cryo-EM structure of the chicken FA core complex (PDB: 6SRI, 4.2 Å) revealed an asymmetric structure, where two copies of FANCB and FAAP100 act as the central core for binding of two copies of FANCL [23] (Figure 4A,B). The importance of FANCB as the central core of the complex explains why mutations in the *FANCB* gene are rare and result in severe FA phenotypes [44]. As FANCL is the E3 RING ligase enzymatic component of the complex, essential for *in vitro* transfer of ubiquitin by the E2 enzyme FANCT (also called UBE2T), the presence of two FANCL molecules raised speculation on mechanisms of ubiquitin transfer. Given that the substrate of monoubiquitination is also dimeric, it was hypothesized that two copies of FANCB:FAAP100:FANCL complex might coordinate ubiquitin conjugation onto each subunit – one FANCL for FANCD2 monoubiquitination and the other FANCL for FANCI monoubiquitination [39]. However, the cryo-EM structure of human FA core complex bound to FANCT resolved up to 3.1 Å (not yet available in PDB, so shown as a cartoon in Figure 4C), revealed that only one of the FANCL RING domains is engaged by the E2 enzyme [43]. The other FANCL RING is buried within a complex of FANCC, FANCE and FANCF.

This binding of one FANCL by the FANCC:E:F module creates an unusual asymmetry within the FA core complex that underscores how this module can pin FA core complex to a preferred orientation on the substrate [43]. In addition, FANCE binds directly to FANCD2 [43,45]. The other copy of FANCL interacts with FANCT, first for FANCD2 monoubiquitination, and then FANCI monoubiquitination. This sequential monoubiquitination reaction fits with the observed biology: FANCD2 modification precedes that of FANCI both *in vitro* [17] and *in vivo* [46].

Overall, the new structures support many genetic and biochemical experiments that pointed to segregation of the FA core complex into three independent subcomplexes, FANC**B**–FANC**L**–FAA**P100**, FANC**C**–FANC**E**–FANC**F** and FANC**A**–FANC**G**–FAA**P20** [17,47,48]. Not yet discussed, and most enigmatic, is the role played by the FANCA-G-p20 complex. FANCA is the largest protein in the complex, and mutation in *FANCA* accounts for more than 65% of FA patients in the U.S.A. and Europe [16]; FANCG contains multiple tetratricopeptide repeat motifs (TPRs) that act as scaffolds in multiprotein complexes [49] and FAAP20 is a small protein made up almost entirely of a UBZ-like ubiquitin binding domain [50]. The FANCA-G-p20 module is essential for FANCI–FANCD2 ubiquitination *in vivo* [51] but appears to play no role *in vitro* [17]. Unlike the single copy of FANCC-E-F within the FA core complex, there are two copies of FANCA-G-p20. One of these copies was not observed in the cryo-EM structure of the chicken FA core complex (Figure 4A,B), however two separate structures of just the FANCA-G module and FANCA CTD dimer reveal a novel asymmetric dimerization occurs in FANCA module (PDB: 6LHV and 6LHS, resolution 4.59 and 3.35 Å [22], (Figure 4D,E), which is maintained in the structure of the full human FA core complex [43].

Dimerization of the FANCA-G module forms independently of the dimerization of the FANCB-L-p100 module. It is mediated through an interface between two FANCA molecules, at the middle of the arc-shape CTD of FANCA, which acts as a scaffold for the module in a ‘head to tail’ manner (Figure 4C). Consistent with the overall asymmetry of the FA core complex, each FANCG molecule binds to each FANCA, and the rest of the FA core complex differently [22]. The first FANCA molecule binds to the first FANCG through its C-terminal end [22]. This FANCG engages a WD40 domain in FAAP100, and two conserved loops in FANCF, on the inactive side of the full FA core complex [43]. The second FANCA molecule binds to the second FANCG through its NTD, via an entirely different interaction interface (Figure 4D). This second FANCG also contacts the FA core complex through a novel interface, in direct association with two separate regions of FANCB – this time on the active side of the complex. The multiple interaction points between FANCA and FANCG, and other FA complex proteins, may help explain why some FA-associated mutations can cause disassembly of the FA core complex, but not loss of interaction between individual subunits [52]. It is presently not clear how all of these unusual associations are established, beyond the observation that the FANCC-E-F module (as a monomer) can block certain conformations and interactions that would otherwise lead to a symmetric complex [23].

So, what *is* the role of FANCA-G-p20? In *in vitro* experiments, FANCA forms a homodimer to promote ssDNA annealing and ssDNA exchanging [53]. The structure of isolated FANCA:FANCG complex does indeed reveal a region of the protein that contains a positively charged surface that appears to fit the criteria of an ssDNA channel leading to a region of annealing [22]. This module therefore plays an unconventional role in FA pathway rather than just to facilitate FANCD2 monoubiquitination. But it is not clear how this function is coupled to the activation of monoubiquitination, and much of the evidence suggests it is not coupled [53]. Instead, the presence of the FANCA-G-p20 within the FA core complex has been demonstrated to (a) stabilize its asymmetric architecture [23], (b) promote its nuclear localization [52] and (c) activate its chromatin retention.

### Which structures are still required, and what will the future hold?

The combined effort of a few laboratories in a short space of time has uncovered so many fascinating and unexpected discoveries related to the structures of FA proteins. A structural investigation of FANCI^Ub^–FANCD2^Ub^ bound by a ubiquitin-binding substrate would help identify the further significant changes required in overall architecture in support of the ‘FANCI–FANCD2 direct-recruitment of repair factors to DNA damage sites’ model that has long been proposed. Alternatively, there might be no true direct-recruitment and instead, a structure of filamentous FANCI^Ub^–FANCD2^Ub^ on longer DNA might indicate how such arrays protect DNA during replication fork stalling at interstrand cross-links and R-loops. For the substrate, mechanistic insight might also come from a co-structure of USP1:UAF1 and FANCI–FANCD2^Ub^, which would uncover how the important deubiquitination reaction is controlled.

The *tour-de-force* investigations of the FA core complex still lack a mechanistic explanation for how FANCI is monoubiquitinated subsequent to FANCD2, and also do not describe the role of targeting and activation of the enzyme to specific locations in DNA. However, the high resolution maps do give us an exciting opportunity to develop structure-guided inhibitors or activators of the FA pathway, that have long been proposed as chemosensitizers or even stand-alone single agents in the treatment of cancer and other proliferation disorders [10,54,55]. The important structures of FANCI^Ub^–FANCD2^Ub^ and the FA core complex present many more opportunities for FA to become a research groundbreaker.

## Summary

- New cryo-EM structures of key proteins and steps in the FA pathway will guide our understanding of DNA repair and the role of specific proteins and mutations in FA and cancer.
- Structure of monoubiquitinated FANCI–FANCD2 reveals DNA clamping, which is proposed to be a critical determinant of DNA repair.
- Structure of the FA core complex reveals how the asymmetry of the complex is established, and how different subunits participate in the monoubiquitination of FANCD2.
- In addition to monoubiquitination, the FANCA:FANCG:FAAP20 subcomplex plays an unconventional role in the FA pathway.

## Open Access

Open access for this article was enabled by the participation of The University of Melbourne in an all-inclusive *Read & Publish* pilot with Portland Press and the Biochemical Society under a transformative agreement with CAUL.

## Abbreviations

CTD: C-terminal domain
dsDNA: double-stranded DNA
EM: electron microscopy
FA: Fanconi anemia
NTD: N-terminal domain
TPR: tetratricopeptide repeat motif
